# Acoustic and Categorical Dissimilarity of Musical Timbre: Evidence from Asymmetries Between Acoustic and Chimeric Sounds

**DOI:** 10.3389/fpsyg.2015.01977

**Published:** 2016-01-05

**Authors:** Kai Siedenburg, Kiray Jones-Mollerup, Stephen McAdams

**Affiliations:** ^1^Centre for Interdisciplinary Research in Music Media and Technology, Schulich School of Music, McGill UniversityMontreal, QC, Canada; ^2^Signal Processing Group, Department of Medical Physics and Acoustics and Cluster of Excellence Hearing4All, University of OldenburgOldenburg, Germany

**Keywords:** timbre perception, dissimilarity ratings, categorization, auditory representation, acoustic modeling

## Abstract

This paper investigates the role of acoustic and categorical information in timbre dissimilarity ratings. Using a Gammatone-filterbank-based sound transformation, we created tones that were rated as less familiar than recorded tones from orchestral instruments and that were harder to associate with an unambiguous sound source (Experiment 1). A subset of transformed tones, a set of orchestral recordings, and a mixed set were then rated on pairwise dissimilarity (Experiment 2A). We observed that recorded instrument timbres clustered into subsets that distinguished timbres according to acoustic and categorical properties. For the subset of cross-category comparisons in the mixed set, we observed asymmetries in the distribution of ratings, as well as a stark decay of inter-rater agreement. These effects were replicated in a more robust within-subjects design (Experiment 2B) and cannot be explained by acoustic factors alone. We finally introduced a novel model of timbre dissimilarity based on partial least-squares regression that compared the contributions of both acoustic and categorical timbre descriptors. The best model fit (*R*^2^ = 0.88) was achieved when both types of descriptors were taken into account. These findings are interpreted as evidence for an interplay of acoustic and categorical information in timbre dissimilarity perception.

## 1. Introduction

Timbre is often considered as one of the “last frontiers” in auditory science. Leaving aside the general agreement that a definition by negation (ANSI, 1994) is unsatisfactory (Krumhansl, 1989; Bregman, 1990; Hajda et al., 1997), the notion is usually understood in a 2-fold manner. Timbre first denotes that auditory attribute that lends sounds a sense of “color.” This quality emerges from a number of acoustic cues—perceptually integrated into a *timbral Gestalt*—the most important of which include the spectral envelope shape, attack sharpness, spectrotemporal variation or modulation, roughness, noisiness, in addition to features that may be idiosyncratic to certain instruments (McAdams, 2013). For acoustic instruments, this bundle of features usually covaries with pitch register and dynamics or playing effort (Handel and Erickson, 2001). At the same time, timbre allows for the categorization of sound sources (McAdams, 1993; Pressnitzer et al., 2013) and for the inference of the mechanics of sound-producing objects and events (Giordano and McAdams, 2010). This gives rise to a cognitive representation of a sound in terms of its source-cause properties that can remain invariant across drastic changes in the acoustic signal (Handel, 1995).

Most cornerstones of the perceptual representation of musical timbre are based on dissimilarity ratings: Two tones are presented in succession per experimental trial, and listeners rate their degree of dissimilarity, such that the task does not require any verbal labeling of sounds. Starting with the early work of Plomp (1970), Wessel (1973), and Grey (1975), multidimensional scaling (MDS, see Kruskal, 1964; Winsberg and De Soete, 1993) has been the most important tool for the analysis of the resulting dissimilarity data. Its basic idea is to yield a spatial configuration of the rated stimuli, the *timbre space*, in which spatial distance corresponds to rated dissimilarity. The space is spanned by the rating data's latent dimensions which can be interpreted psychophysically by correlation with continuous acoustic descriptors. For example, McAdams et al. (1995) presented a three-dimensional solution including values for dimensions or features specific to each sound, as well as weights on shared dimensions and specificities for latent classes of subjects. The first spatial dimension correlated with (log-) attack time (AT), the second with the spectral center of gravity (SCG), the third with spectral variation over time (“spectral flux”). SCG and AT have been confirmed to be perceptually salient in a number of studies (Lakatos, 2000; Halpern et al., 2004; Caclin et al., 2005). Recently, Elliott et al. (2013) used high-dimensional modulation spectra that represent a signal's joint spectro-temporal variability, followed by methods of dimensionality reduction in order to provide an acoustic basis for the five-dimensional MDS space they had obtained. They observed that the approach has similar predictive power compared to an acoustic description based on scalar audio descriptors (including measures such as spectral and temporal center of gravity).

Two implicit assumptions of this approach deserve further notice. First, dissimilarity ratings are symmetric (none of the mentioned studies tested this empirically). Second, dissimilarity ratings are based on the sounds' acoustic properties and are not related to source categories or semantic associations (none of the above-mentioned studies reported having specifically instructed participants to rate acoustic quality). The goal of the present paper is to demonstrate that there are cases under which these subtle but important assumptions can fail.

In order to provide some background, we first review previous work on timbre similarity and categorization. We then outline a related controversy on the continuous or categorical nature of psychological similarity, exploring more deeply the conditions under which asymmetric similarities are likely to occur. Note that this work concerns the role of familiar instrument categories in dissimilarity ratings; we will not address *categorical perception* of timbre in the sense of differential inter- and intra-category discriminability of stimuli (see Donnadieu, 2008).

### 1.1. Sound source categories and similarity

Regarding the inference of material and excitation properties, listeners have been shown to reliably infer geometry and material properties such as damping of sounding objects (McAdams et al., 2004, 2010; Giordano and McAdams, 2006, 2010; Giordano et al., 2010b). However, acoustic cues used for dissimilarity rating and categorization partially differed (McAdams et al., 2010). Research with vocal and instrumental timbres has demonstrated that neither solely spectral, nor solely temporal cues are sufficient to account for timbre categorization (Agus et al., 2012). Curiously, Suied et al. (2014) highlighted in a subsequent study that acoustic cues for timbre categorization may reside on very short time-scales, i.e., likely in the spectral domain. Using gated vocal and instrumental sounds, listeners could reliably categorize sounds of gate durations as short as around 8 ms. Taken together, these diverse findings suggest that the perceptual system might exploit sensory cues in an opportunistic fashion. Rather than always using the same fixed set of acoustic cues, only the most informative cues are employed with respect to the scenario of a particular perceptual task (also see McAdams et al., 2010; Suied et al., 2014).

Coming back to the similarity rating task, Lakatos (2000) used a set of harmonic instrumental sounds, percussive sounds, and a mixed set to explore MDS and clustering solutions of dissimilarity ratings. As acoustic complexity of sounds increased, in particular for the set of percussive sounds, listeners' responses were interpreted to rely more on categorical representations. Accordingly, Lemaitre et al. (2010) proposed to distinguish between acoustical sound similarity (cognitively represented by auditory sensory representations), causal similarity (represented via the shared and distinct features of the perceptually inferred source-cause mechanisms), and semantic similarity (related to associated meaning or knowledge about the underlying sound event; see Slevc and Patel, 2011, for a more general discussion of semantics in music). Halpern et al. (2004) compared musicians' dissimilarity of heard and imagined musical instrument tones while recording functional magnetic resonance imaging (fMRI). Both conditions presented instrument names visually, and the “heard” condition also presented the instrument's sound. Auditory cortex was active during perception and imagery and behavioral ratings of perceived and imagined dissimilarity correlated significantly (*r* = 0.84). Note that the fMRI data are the only suggestive piece of evidence that there was indeed sensory imagery for timbre, as the correlation could well have been explained by participants comparing non-auditory features of instruments in both tasks, i.e., relying on causal or semantic similarity. In a similar vein, Iverson and Krumhansl (1993) had already found similar MDS solutions for sets of orchestral instrument sounds for which either full tones, only attack portions (80 ms) or only remainders were presented. Giordano and McAdams (2010) presented a meta-analysis of studies on instrument identification and dissimilarity judgments. Instruments were more often confused in identification and rated as more similar when they were members of the same family or were generated by the same manner of excitation (impulsive, sustained), underlining the strong correspondence between continuous sensory and categorical types of timbre similarity.

There still remains the question of whether these links between acoustics and source category are of an intrinsic correlational nature, based on the partial coincidence of acoustic similarity and categories of source mechanics (instruments that feature similar source mechanics will likely feature similar acoustic qualities), or because listeners give significant weight to the causal similarity of stimuli. Most timbre dissimilarity studies have used tones from western orchestral instruments or their synthetic emulations. These are stimuli with which western listeners, whether musicians or non-musicians, inevitably have a lifelong listening experience, and thus can be assumed to possess long-term mental categories (cf. Agus et al., 2010). For unaltered instrumental tones, it thus seems hard to experimentally disentangle acoustic and categorical factors. An example of such a dissociation was nonetheless given by Giordano et al. (2010a), albeit not for timbre specifically. These authors outlined how processing strategies may differ across sound categories: sounds from non-living objects are sorted mainly based on acoustic criteria, whereas the evaluation of living sounds is biased toward semantic information that is partially independent of acoustic cues. The interplay of affordances for source identification and listening experience was further studied by Lemaitre et al. (2010). They observed that sounds with low *causal uncertainty* (measuring the amount of reported alternative causes for a sound) tended to be classified on the basis of their *causal similarities* (i.e., based on source-cause properties), whereas sounds with high causal uncertainty were rather grouped on the basis of acoustic cues. Moreover, so-called “expert listeners” (i.e., musicians, sound artists, sound engineers, etc.) tended to rely more heavily on acoustic cues than non-experts when categorizing sounds with low causal uncertainty.

### 1.2. Similarity and categorization

The previous observations on timbre are surrounded by a long-lasting debate on the nature of perceptual dissimilarity. One basic question is whether similarity is best described by continuous multidimensional spaces or via set-theoretic models based on categorical stimulus features (cf. Tversky, 1977; Shepard, 1987; Ashby, 1992; Tenenbaum and Griffiths, 2001; Goldstone et al., 2015).

Classic work in cognitive psychology shows that for complex, semantically loaded stimuli, geometric reasoning about psychological similarity may be inadequate. In a pioneering paper, Rosch (1975) presented asymmetric data of psychological similarity. Subsequently, Tversky (1977) developed a similarity model based on categorical *features*, binary attributes that a stimulus may or may not possess. Tversky also attacked the symmetry assumption: “Similarity judgments can be regarded as extensions of similarity statements, that is, statements of the form “a is like b.” Such a statement is directional […]. We tend to select the more salient stimulus, or the prototype, as a referent, and the less salient stimulus, or the variant, as a subject. […] We say “North Korea is like Red China” rather than “Red China is like North Korea” (Tversky, 1977, p. 328). He provided a variety of asymmetric empirical data in which the similarity of a prototypical stimulus to a variant was smaller than the reverse.

Shepard (1987) commented that the observed problems of spatial models might only concern stimuli with highly separable perceptual dimensions that do not interfere with each other in perceptual processing. Nonetheless, the results by Melara et al. (1992) seem to render this hypothesis unlikely. Their subjects rated the pairwise similarity of sets of stimuli with varying separability of perceptual dimensions. A perceptually separable audio-visual condition presented stimuli varying in pitch accompanied by visually presented crosses with varying positions. A perceptually integral condition presented auditory stimuli varying in pitch and loudness. For both conditions, a first group of subjects was instructed to judge similarity on the basis of the overall *Gestalt*, another to attend to all perceptual dimensions separately. Data from the latter group were best fitted by a cityblock metric (additive sum of the individual dimensions), whereas dissimilarities from the group that applied a holistic strategy were best approximated by a Euclidean metric (a nonlinear combination of the dimensions). The malleability of ratings, easily modified by instructions, therefore led the authors to conclude that direct similarity ratings involve an interplay of *optional* and *mandatory* perceptual processes. Mandatory processes refer to hard-wired perceptual processes where the weighting of stimulus dimensions is thought not to be under direct control of subjects (Shepard, 1987). Optional processes were interpreted to give subjects a choice of what stimulus facets to attend to and rate. Importantly, Melara et al. (1992) observed both kinds of processes for all stimulus sets they tested, even those classically considered as integral.

Perceptual dimensions of timbre have been described as interactive (Caclin et al., 2007). The above considerations thus suggest that optional processes are likely to be at play, particularly so if sounds can be easily identified or possess heterogeneous semantic affordances. On the other hand, if participants exclusively relied on a stimulus's sensory representation, rating asymmetries should not occur.

### 1.3. The present study

For circumventing the co-occurrence of acoustic similarity and source categories, we chose to compare musicians' dissimilarity ratings of familiar acoustic and unfamiliar synthetic tones specifically generated for the study. We first created timbral transformations that partially preserved the acoustic properties of a set of recorded orchestral instruments (similar to Smith et al., 2002) and let musicians identify and rate the subjective familiarity of the sounds (Experiment 1). The 14 transformations rated as most unfamiliar were then selected for comparison with the 14 recorded acoustic instrumental tones. In Experiment 2A, we then collected dissimilarity ratings for the set of recorded tones, transformed tones, and a mixed set (methodically similar to Lakatos, 2000). We were interested in observing the relation of instrument categories and acoustic similarity in the clustering of the dissimilarity data, as well as potential category-based asymmetries in dissimilarity ratings. We hypothesized that if asymmetries would occur, they would most likely be found between recorded acoustic tones and synthetic transformations, i.e., in the mixed set. Such mixed pairs feature a particularly strong categorical dissimilarity, because one sound is acoustic and the other synthetic, and because there is a gap in familiarity between these two classes of sounds (experimentally controlled by virtue of Experiment 1). We finally conducted an exploratory regression analysis that enabled us to trace out the role of categorical factors for the set of recordings and the mixed set in more detail.

## 2. Experiment 1: identification and familiarity

This experiment was conducted in order to provide a basis for the selection of unfamiliar stimuli without readily available source-cause associations for Experiment 2.

### 2.1. Methods

#### 2.1.1. Participants

There were 15 participants (nine male, six female) with ages between 18 and 36 (*M* = 22.2, *SD* = 4.6). They had a mean of 9.4 years of musical instruction (*SD* = 3.5) and a mean of 5 years experience playing in ensembles (*SD* = 2.9). Two reported possessing absolute pitch. Participants were compensated for their time.

#### 2.1.2. Stimuli and presentation

Stimuli consisted of 14 recordings of single tones from common musical instruments and 70 tones that were derived by digital transformation of the 14 acoustic tones. The recorded timbres consisted of the bass clarinet (BCL), bassoon (BSN), flute (FLT), harpsichord (HCD), horn (HRN), harp (HRP), marimba (MBA), piano (PNO), trumpet (TRP), bowed violoncello (VCE), violoncello pizzicato (VCP), vibraphone (VIB), bowed violin (VLI), and violin pizzicato (VLP), all played at mezzo-forte without vibrato. Piano and harpsichord samples were taken from Logic Professional 7; all other samples came from the Vienna Symphonic Library (http://vsl.co.at, last accessed April 12, 2014). The audio sampling rate used throughout this study was 44.1 kHz. Sounds had a fundamental frequency of 311 Hz (E♭4), and only left channels were used. According to the VSL, the samples were played as 8th-notes at 120 beats per minute, i.e., of 250 ms “musical duration.” However, actual durations varied and were slightly longer than 500 ms for all sounds, such that we used barely noticeable fade-outs of 20 ms duration (raised-cosine windows), in order to obtain stimuli of uniform duration (500 ms). Peak amplitude was normalized across all sounds. This set of 14 timbres is hereafter referred to as “recordings.”

A second set of timbres was generated digitally. The goal was to obtain stimuli for which associations of an underlying source were not readily available and that possessed a reduced degree of perceptual familiarity. At the same time, these stimuli should not differ too strongly in their overall acoustic variability compared to the set of original recordings. We thus decided to digitally transform the spectro-temporal envelopes and acoustic fine structures of the recordings, a procedure that was demonstrated to yield altered (“chimæric”) perceptual properties for speech signals (Smith et al., 2002). Any novel sound was derived from a source signal (“chimæra-source” or “c-source”), the spectrotemporal fine structure of which was amplitude modulated by the spectrotemporal envelope of a second signal that acted as a time-varying filter (“c-filter”). These abbreviations will be used in the rest of this paper in order not to confuse this specific approach with the general technique of source-filter synthesis. More specifically, chimeras were generated in MATLAB version R2013a (The MathWorks, Inc., Natick, MA). Sound signals were decomposed by a linear 24-band Gammatone-filterbank (Patterson et al., 1992) as implemented in the MIRtoolbox (Lartillot and Toiviainen, 2007). Amplitude-envelopes were extracted for every filterband of both c-sources and c-filters, using low-pass filtering and half-wave rectification (Lartillot and Toiviainen, 2007). For every band, the c-filter's envelope values were then imposed onto the c-source by normalizing the c-source's filterband envelopes, followed by point-wise multiplication with the c-filter's time-varying envelope magnitudes. The resulting signal hence possessed the spectrotemporal envelope of the c-filter and the fine structure of the c-source (cf. Smith et al., 2002).

We chose to use three different types of sounds to act both as c-sources and c-filters. The first type consisted of the fourteen recordings mentioned above. Sounds of the second type (conceived to further decrease perceptual familiarity) were generated in four steps: We (i) decomposed the acoustic sounds into twenty-four Gammatone-filterbands, (ii) randomly selected four sounds from the fourteen, (iii) allocated their filterbands such that each of the four sounds contributed to the new sound with six different bands, and (iv) added all twenty-four distinct bands. This process is called “filterband scrambling” (FBS) hereafter. Six such sounds were selected, denoted as FBS 1–6 below. Among these, FBS 1&2 possessed a slow attack, FBS 3&4 a sharp attack, and FBS 5&6 attacks in between the two extremes. The third type of sounds simply consisted of a zero-phase harmonic tone complex with a fundamental frequency of 311 Hz. Note that on their own, type one should be highly familiar to participants, and type two should be less familiar. Despite its artificiality, the harmonic tone complex may be familiar due to its status in electronic music. If taken as c-filter, the harmonic tone complex has a neutral effect due to its flat spectral envelope, i.e., coincides with no envelope filtering at all. Using sounds of type one as c-filter should affect familiarity of recordings acting as c-sources, as spectrotemporal envelope properties are substantially altered. This provided 21 (14 + 6 + 1) distinct sounds in total. Any possible combination of c-sources and c-filters was then used to generate 441 (21 × 21) chimaeric signals, 70 of which were pre-selected manually for the experiment. The selection was subject to the constraint that every c-source and c-filter signal was required to be selected at least once; for recordings acting as c-filters, each c-filter was selected at least twice. Additionally, the selection favored timbres that seemed unfamiliar to the experimenters, but did not contain too much narrowband noise (an artifact that was introduced in some transformations by boosting the amplitude of filterbands with low energy). All sounds were normalized in peak amplitude. See the Supplementary Material for sound examples.

#### 2.1.3. Procedure

The research reported in this manuscript was carried out according to the principles expressed in the Declaration of Helsinki and the Research Ethics Board II of McGill University has reviewed and approved this study (certificate # 67-0905).

Participants first completed a standard pure-tone audiogram to ensure normal hearing with hearing thresholds of 20 dB HL or better with octave spacing in the range of 250–8000 Hz (ISO 398-8, 2004; Martin and Champlin, 2000). In every trial of the experiment, a single stimulus from the 70 transformations and 14 recordings was presented to participants. They were asked to choose an identifier from a list of eight possible options. The list consisted of six musical instrument names. For recorded timbres, it contained the correct label and five randomly chosen labels from the remaining set. For transformations, it involved the two labels of the timbres that had been involved as c-source and c-filter, plus four labels chosen randomly from the remaining set. For instance, if a transformation was derived from a piano as a c-source, whose time-varying spectral envelope was exchanged with that of a violin, then both instrument names, piano and violin, would be part of the list. The list further contained the two options “unidentifiable” and “identifiable but not contained in list.” If the participant selected the latter option, a dialogue box appeared prompting them to enter an appropriate identifier in the text box on screen. They could then continue, whereupon they heard the sound a second time and were presented with two analog-categorical scales on which they had to rate familiarity (1-highly unfamiliar, 5-highly familiar) and artificiality (1-very natural, 5-very artificial). Sounds were presented in randomized order. Three example trials preceded the 84 experimental trials. The full experiment took around 45 min.

Experiments took place in a double-walled sound-isolation chamber (IAC Acoustics, Bronx, NY). Stimuli were presented on Sennheiser HD280Pro headphones (Sennheiser Electronic GmbH, Wedemark, Germany), using a Macintosh computer with digital-to-analog conversion on a Grace Design m904 (Grace Digital Audio, San Diego, CA) monitor system. The experimental interface and data collection were programmed in the Max/MSP audio software environment (Cycling 74, San Francisco, CA). The average presentation level was 78 dB SPL (range = 75–82 dB SPL) as measured with a Brüel and Kjær Type 2205 sound-level meter (A-weighting) with a Brüel and Kjær Type 4153 artificial ear to which the headphones were coupled (Brüel and Kjær, Nærum, Denmark).

### 2.2. Results

By construction, correct responses for the identification task only existed for the recordings. Here, correct identification rates ranged from 0.46 (BCL and BSN) to 1.0 (TRP). The mean identification rate for all 14 recordings was 0.73 (*SD* = 0.180) with chance baseline equal to 1∕8 = 0.125. The bass-clarinet (BCL) was the only recording for which an alternative category, “unidentifiable,” was selected most often (0.53).

From the remaining 70 transformations, 29 were most often identified as other musical instruments (i.e., the category that was selected by the majority of subjects) with average selection rates of 0.47 (*SD* = 0.12). From these 29 transformations, the category chosen most often for 23 sounds was an instrument that acted either as c-source or c-filter in its generation. Only six transformations failed to be related to their source or filter by a majority of participants; these were the transformations BCL-VLP (→ heard as VIB; BCL denoting c-source, VLP c-filter), VIB-BCL (→ MBA), VLI-BSN (→ HRN), VCP-FBS (→ MBA), FBS-FBS (→ VIB), and FBS (→ MBA). Thirteen transformations were most often selected as “unidentifiable” with stimulus-wise mean selection rates of 0.55 (*SD* = 0.21). Twenty-eight transformations were selected as “identifiable, but not in the list” with mean selection rates of 0.55 (*SD* = 0.16). If subjects had selected the latter category, they were asked to briefly describe what they had heard in a written response. Three different types of responses appeared most often here: 41% of these responses mentioned single orchestral instruments; 37% mentioned a mix of multiple instruments (e.g., “piano and trombone in unison”); 16% mentioned electronic means of sound synthesis; 6% were hard to categorize (e.g., participant 7: “Ahh yes patch 87: plucking a frog.”).

Pearson correlations between the proportion of “unidentifiable” votes per stimulus and mean familiarity ratings were strong and negatively associated, *r*_(82)_ = −0.88, *p* < 0.001, as was the correlation between familiarity and artificiality, *r*_(82)_ = −0.86, *p* < 0.001. The harmonic tone complex without filtering obtained maximal artificiality ratings (*M* = 4.95, *SD* = 0.10) and medium familiarity (*M* = 3.37, *SD* = 1.34) and was an obvious outlier in the latter correlation; removing this datum increased the correlation to *r*_(81)_ = −0.89.

**Figure 1:**
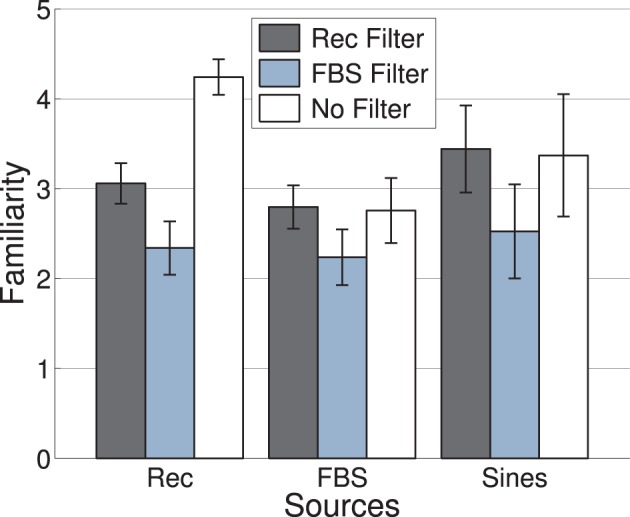
**Experiment 1: Mean familiarity of signals generated by nine different combinations of c-source (x-axis) and c-filter (color-coded), see text for a description of c-source and c-filter**. Error bars represent 95% confidence intervals.

Mean familiarity ratings as a function of c-source and c-filter are displayed in Figure 1. Given that the pre-selection of stimuli attempted to select unfamiliar timbres, a causal interpretation of effects of c-source and c-filter on familiarity would not be appropriate. It should be remarked, however, that the highest familiarity ratings were as expected obtained by the non-filtered recordings. At the same time, the filterbank scrambled signals (FBS) acting as c-filters achieved the lowest average familiarity ratings for all c-sources.

### 2.3. Discussion

The identification scores for the 14 recorded timbres yielded correct choices by the majority of subjects for all instruments except the bass-clarinet, for which “unidentifiable” took the lead. Apart from this one exception which also possessed the lowest familiarity ratings among the 14 unaltered timbres (familiarity and rates of “unidentifiable” choices were strongly correlated), results indicated that musicians were able to identify acoustic timbres of less than 500 ms duration from a single presentation. Yet, the current data exhibit considerable variance in the percentage of correct identifications across different instruments (ranging from 46 to 100%), a finding that parallels the divergent estimates of identification accuracy in the literature (Srinivasan et al., 2002). From the 70 transformations, the majority vote identified 29 as alternative instruments that were provided in the list of options. Among these 29, around 80% were correctly identified as instruments that had either acted as c-source or c-filter in the synthesis process. This underlines musicians' abilities to identify sound source properties and mechanics (Giordano and McAdams, 2010), even in situations where these are severely altered.

Familiarity ratings and the proportion of “unidentifiable” votes were strongly correlated. Familiarity and artificiality ratings shared around 77% of mutual variance if one outlier was removed. The most likely factor that may have caused this strong correlation could be the digital transformation used in the production of stimuli. The more impact the transformation had on the original signal structure, the less familiar the resulting timbres appeared to be. However, the plain harmonic tone complex received the highest artificiality ratings, while far from being rated as least familiar. The fact that this signal did not follow the overall trend suggests that not any digitally synthesized tone obtains low familiarity ratings, which justifies our usage of a somewhat elaborate signal transformation. Not surprisingly, the highest familiarity ratings were obtained for the unaltered recordings.

## 3. Experiment 2: timbre dissimilarity of acoustic recordings and synthetic transformations

Experiment 1 suggested that overtly simple means of sound synthesis may fail to create tones that are unfamiliar to musicians, but confirmed that familiarity and source identifiability were highly related in the presented set of recordings and transformations. In order to study the role of sound categories and familiarity in dissimilarity perception, we selected 14 transformations rated as least familiar in Experiment 1 and used them together with a set of recordings in a dissimilarity rating task for musicians. Due to the strong correlation of familiarity and identifiability, the selected transformations consequently only scarcely afforded unambiguous identification of source-cause categories. We were interested in the ways in which the dissimilarity structures would be affected by categorical properties of tones, such as instrument families within the set of recordings, and whether asymmetries would occur between synthetic and acoustic tones.

Specifically, we collected dissimilarity ratings for the set of recordings (Set 1), transformations (Set 2) and a mixed set (Set 3). In Experiment 2A, the order of the presentation of tones within a pair was counterbalanced across (musician) participants. Using a within-subjects design, Experiment 2B was conducted in order to confirm rating asymmetries in Set 3 from Experiment 2A; a new group of musicians rated both orders of presentations of only the mixed set of tones (Set 3). For the sake of brevity, methods and results of both experiments will be described in the same section below.

### 3.1. Methods

#### 3.1.1. Participants

##### 3.1.1.1. Experiment 2A

Twenty-four musicians (11 male, 13 female) with ages between 18 and 36 years (mean age = 24.1, *SD* = 5.3) took part. Participants had a mean of 12.8 years of musical instruction (*SD* = 6.4) and a mean of 7.3 years experience playing in ensembles (*SD* = 4.6). One participant reported possessing absolute pitch. Participants were compensated for their time.

##### 3.1.1.2. Experiment 2B

Twenty-four musicians (10 male, 14 female) with ages between 18 and 28 years (mean age = 22.5, *SD* = 2.7) participated. They had a mean of 11.1 years of musical instruction (*SD* = 3.7) and a mean of 6.3 years experience playing in ensembles (*SD* = 3.6). Seven participants reported possessing absolute pitch. Participants were compensated for their time.

#### 3.1.2. Stimuli and presentation

##### 3.1.2.1. Experiment 2A

In every trial, pairs of timbres of 500-ms duration each were presented with a 300-ms inter-stimulus interval. Stimuli consisted of the 14 acoustic recordings (Set 1) and 14 transformed sounds (Set 2) that had obtained the lowest familiarity ratings in Experiment 1. A mixed set contained the seven most familiar recordings and the seven least familiar transformations (Set 3). All stimuli had a 311 Hz fundamental frequency. Table 1 list all stimulus names, labels, and their mean familiarity ratings from Experiment 1 for recorded and transformed stimuli, respectively. Stimuli included in Set 3 are indicated with asterisks.

**Table 1:** **List of recordings and transformations used in Experiments 2A and 2B with mean familiarity ratings**.

**No. of**	**Set 1 (Recordings)**						**Set 2 (Transformations)**			
	**Instrument**	**Label**	**Famil**.	**Source**	**Filter**	**Label**	**Famil**.
1	Bass Clarinet	BCL^*^	4.3	Bass Clarinet	FBS2	BCL-FBS2^*^	1.6
2	Bassoon	BSN	3.1	Bassoon	Harpsichord	BSN-HRP^*^	1.9
3	Flute	FLT	4.1	FBS1	Violoncello	FBS1-VCE^*^	1.8
4	Harpsichord	HCD^*^	4.5	FBS2	Violoncello	FBS2-VCE	2.1
5	Horn	HRN	4.2	FBS3	FBS2	FBS3-FBS2	2.1
6	Harp	HRP	4.1	FBS6	Trumpet	FBS6-TRP^*^	1.9
7	Marimba	MBA^*^	4.6	Flute	FBS1	FLT-FBS1	2.1
8	Piano	PNO	4.3	Harp	FBS3	HRP-FBS3^*^	1.7
9	Trumpet	TRP^*^	4.8	Harpsichord	FBS4	HRP-FBS4	2.3
10	Violoncello	VCE^*^	4.7	Horn	FBS6	HRN-FBS6^*^	2.0
11	Violoncello Pizz.	VCP^*^	4.5	Marimba	Harpsichord	MBA-HRP	2.0
12	Vibraphone	VIB	4.3	Trumpet	FBS5	TRP-FBS5	2.3
13	Violin	VLI	3.4	Violin	Piano	VLP-PNO	2.4
14	Violin Pizz.	VLP^*^	4.4	Violoncello	Vibraphone	VCE-VBS^*^	2.0

Labels with asterisks (

* *) indicate timbres that were also used in Set 3*.

Six expert listeners equalized the perceived loudness of sounds against a reference sound (marimba), using a protocol designed in PsiExp (Smith, 1995) for the music-programming environment Pure Data (http://puredata.info, last accessed April 12, 2014). Stimuli were presented through a Grace m904 amplifier, and listeners used a slider on the computer screen to adjust the amplitude-multiplier of the test sound until it matched the loudness of the reference sound. Loudness was then normalized on the basis of the median loudness adjustments. Both for loudness equalization and the main experiment, the same apparatus was used as in Experiment 1. The average presentation level after loudness normalization was 66 dB SPL (range = 58–71 dB SPL).

##### 3.1.2.2. Experiment 2B

Only the mixed set (Set 3) was used. Otherwise, stimuli were identical to Experiment 2A.

#### 3.1.3. Procedure

##### 3.1.3.1. Experiment 2A

Normal hearing was ensured as in Experiment 1. Subjects were asked to rate the dissimilarity of two successively presented sounds on an analog-categorical scale (a continuous rating scale with marks between 1-identical and 9-very dissimilar at the extremes) by answering the question “How dissimilar are these two sounds?” They were able to hear the pair as many times as desired by pressing a play button, but were encouraged to move at a reasonable pace. Four example trials were given. Before the start of each experimental session, participants heard all sounds from the respective set in random order. The overall experiment consisted of one session per set. The mixed set came last for all participants. For all three sets, each pair was presented once in one order. The order of presentation (AB vs. BA for timbres A and B) was counterbalanced across subjects. Pairs of identical timbres were included, yielding 105 comparisons per set. There was a 10-min break between each set. The full experiment took 1.5–2 h to complete.

##### 3.1.3.2. Experiment 2B

In contrast to Experiment 2A, the full 14 × 14 matrix of pairwise comparisons including both orders of pairs was presented to every subject. This was administered in a single session with 196 trials in fully randomized order, lasting on average less than 40 min.

### 3.2. Results

**Figure 2:**
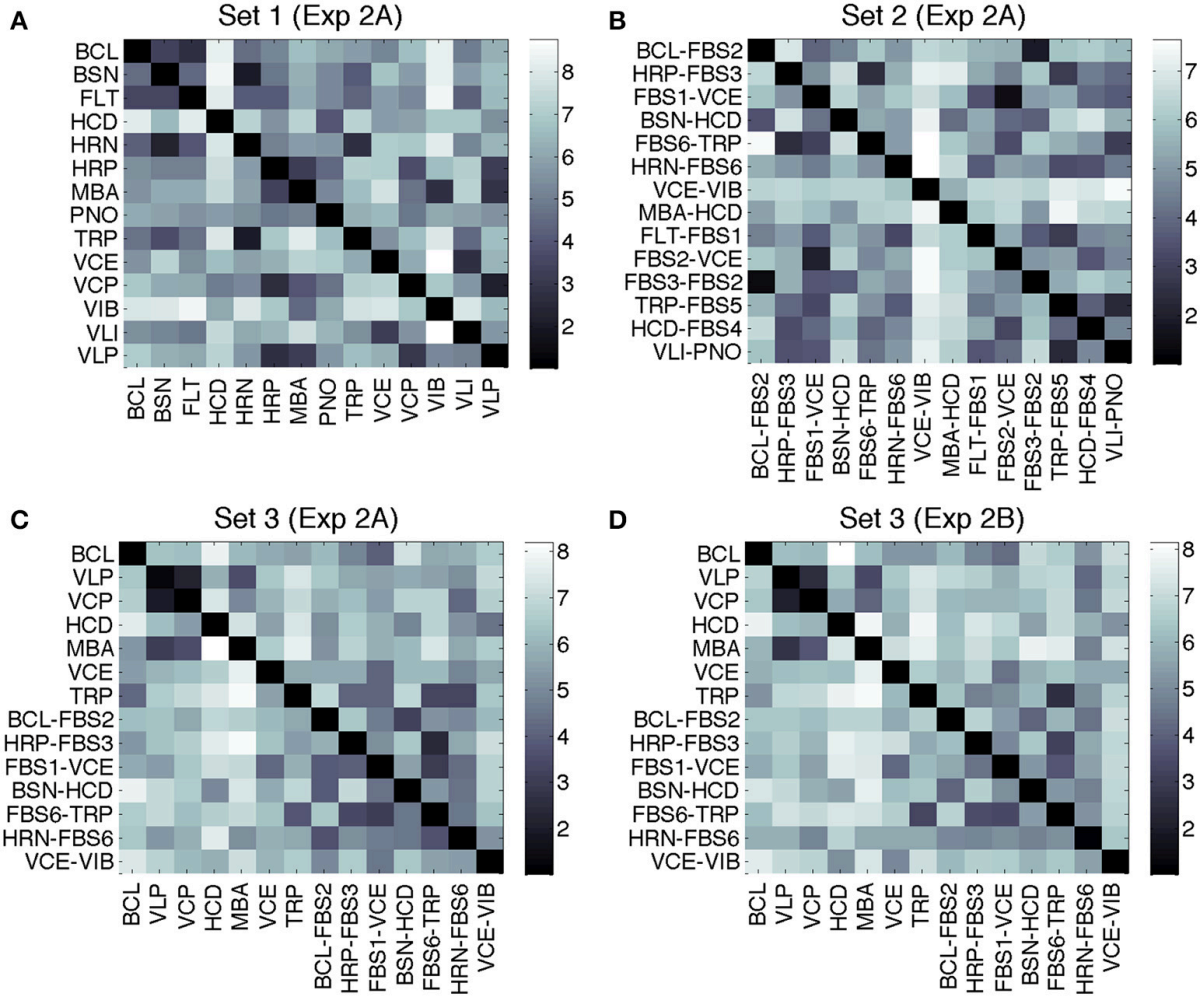
**Mean dissimilarity ratings for Experiment 2A, Set 1 (A), Set 2 (B), Set 3 (C), and Experiment 2B, Set 3 (D)**. Rows determine the first stimulus, columns the second.

Average dissimilarity ratings for Sets 1–3 from Experiments 2A and 2B are displayed in Figure 2. In Experiment 2A, mean ratings were *M* = 5.5 (*SD* = 1.9) for Set 1, *M* = 4.9 (*SD* = 1.7) for Set 2, and *M* = 5.4 (*SD* = 1.7) for Set 3. Mean ratings in Experiment 2B for Set 3 were *M* = 5.7 (*SD* = 1.7). Ratings for Set 3 from Experiments 2A and 2B were highly correlated, *r*_(194)_ = 0.94, *p* < 0.001.

#### 3.2.1. Dissimilarity clusters

**Figure 3:**
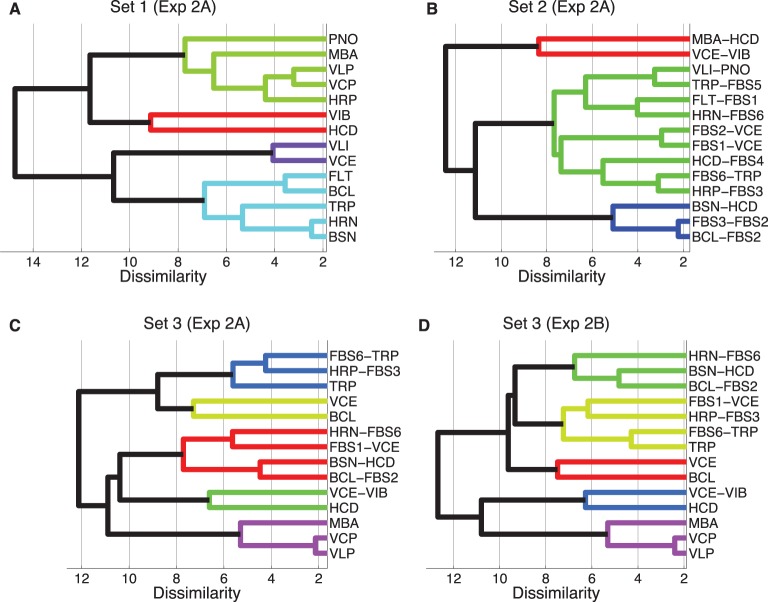
**Hierarchical clustering of mean dissimilarity ratings from Experiment 2A, Sets 1 (A), Set 2 (B), and Set 3 (C), as well as Experiment 2B, Set 3 (D), using the complete-linkage method**. Color-coded groups are specified by a 70% linkage cutoff.

Hierarchical cluster analyses were computed on the basis of dissimilarity data averaged over the directionality of the comparison (symmetry being a condition of the clustering algorithm). This approach admittedly can only serve as a rough approximation for the subset of recordings-transformations from Set 3, as indicated by the analyses on asymmetries below. Figure 3 shows the corresponding clustering trees, using the complete-linkage method. The latter is based on a function that iteratively computes the distance of the two elements (one in each cluster) that are the farthest away from each other. Thresholds for overall grouping (indicated by color-coding in the figure) was 70% of maximal linkage (the default value of the Matlab dendrogram.m function that was used). Sets 1, 2, and 3 yielded 4, 3, and 5 clusters, respectively. The cophenetic correlation coefficients (the linear correlations between the tree solutions and the original dissimilarities) were 0.80, 0.86, and 0.65 for Sets 1–3, respectively, indicating the worst fit for Set 3.

More specifically, the clustering solution for Set 1 partially corresponded to the well-known families of musical instruments: wind instruments clustered together (turquoise), similarly to bowed string instruments (VLI and VCE, violet). The top cluster (green) corresponds to impulsively excited instruments, and there is one cluster with two very bright and impulsive instruments (VIB and HCD, red).

The tree for Set 2 is harder to interpret, due to the lack of definite source categories. Here, only three clusters emerged, one of which contained nine of the 14 timbres. It is further to be noted that the identity of the timbres corresponding to c-sources or c-filters did not seem to play out as a definite predictor for clustering. For instance, although the timbres MBA-HCD and BSN-HCD contain the same c-filter, they turned out to be maximally far apart in the tree. On the other hand, the timbres FBS1-VCE and FBS2-VCE were very close in the tree.

Set 3 yielded a solution with five clusters, from which two were mixed clusters (containing both recordings and transformations), two contained recordings only, and one contained only transformations. From bottom to top, the first cluster (in violet) retained impulsively excited timbres from their cluster in Set 1. The second cluster (green) joined the brightest recordings (HCD) and transformations (VCE-VIB). The largest cluster of this set (red) contained four transformations, two each stemming from relatively close clusters in Set 2. The cluster consisting only of VCE and BCL (bright green) again joined relatively proximal timbres from Set 1. Finally, the last cluster (blue) connected two very similar timbres from Set 2 with a single recording (TRP). The clusters obtained from Experiment 2B (Set 3) were identical apart from the timbre FBS1-VCE.

#### 3.2.2. Asymmetry

Difference matrices for dissimilarity ratings from Experiment 2A were obtained by excluding identical pairs, i.e., the comparisons (A,A), (B,B), etc., and subtracting mean dissimilarity ratings for pairs with reversed order, i.e., dissim(A,B)—dissim(B,A). Specifically, we subtracted the upper from the lower triangular entries of the initial dissimilarity matrices. The values of the resulting triangular difference matrices should be centered at zero, if dissimilarity ratings were symmetric. Shapiro-Wilk tests did not indicate deviations from normality for any of these four difference matrices, all *p* > 0.49. Set 3 contained three types of pairs that were analyzed in their own right: recordings-recordings (RR), transformations-transformations (TT), and recordings-transformations (RT).

**Figure 4:**
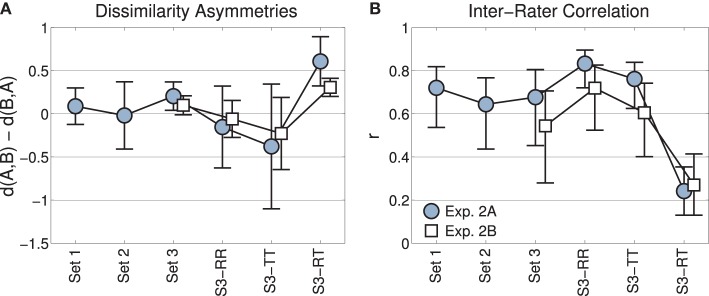
**Experiments 2A and 2B**. **(A)** Mean rating asymmetries across the three sets, and the subsets of Set 3 with the pairs recording-recording (RR), transformation-transformation (TT), recording-transformation (RT). Errorbars indicate 95% confidence intervals. **(B)** Inter-rater agreement as measured by mean Pearson correlation coefficients. Errorbars indicate 95% confidence intervals obtained by bootstrapping.

Figure 4A depicts means and confidence intervals of the corresponding differences data (lower minus upper triangular matrices). The positive mean for the subset of mixed pairs from Set 3 (“S3-RT”) indicates that dissimilarity ratings tended to be greater for transformations followed by recordings (lower triangular matrix) than for recordings followed by transformations (upper triangular matrix). No other (sub)set featured such an asymmetry: after correction for multiple comparisons (using the Bonferroni method, *n* = 6 comparisons, i.e., α_*crit*_ = 0.0083), two-sided single-sample *t*-tests against a mean of zero for difference matrices yielded non-significant results for all sets apart from the subset of mixed (RT) pairs [Set 1: *t*_(90)_ = 1.24, *p* = 0.26, Set 2: *t*_(90)_ = −0.18, *p* = 0.85, Set 3: *t*_(90)_ = 2.12, *p* = 0.037, Set 3-RR: *t*_(20)_ = −0.87, *p* = 0.49, Set 3-TT: *t*_(20)_ = −2.0, *p* = 0.04, Set 3-RT: *t*_(48)_ = 5.3, *p* < 0.001]. This means that only the ratings of mixed pairs exhibited reliable asymmetries. This pattern of results was replicated in Experiment 2B [Set 3: *t*_(90)_ = 1.8, *p* = 0.073, Set 3-RR: *t*_(20)_ = −0.70, *p* = 0.49, Set 3-TT: *t*_(20)_ = −2.2, *p* = 0.08, but Set 3-RT: *t*_(48)_ = 4.3, *p* < 0.001].

#### 3.2.3. Inter-rater agreement

We assessed inter-rater agreement by calculating inter-rater correlations (IRC) for ratings from Sets 1, 2, and 3, as well as the RR, TT, and RT subsets of Set 3. For any such (sub)set of comparisons and N subjects, we obtained the IRC by computing the mean (Fisher-transformed) Pearson correlation coefficients between all N(N-1)/2 pairs of subjects. Mean (back-transformed) IRCs are displayed in Figure 4B with 95% confidence intervals as obtained by bootstrapping (Efron and Tibshirani, 1994). Every bootstrap sample drew 28 comparisons with replacement (the cardinality of the smallest subsets of comparisons, RR and TT, such that comparison of IRC across different (sub-)sets is not confounded by a difference in variable size); we used 1000 random drawings and the percentile method to obtain confidence intervals (Efron and Tibshirani, 1994). Most obviously, mean IRCs for the first five sets are in the range of 0.6–0.8 for Experiment 2A and somewhat lower for Experiment 2B, but not significantly so. However, for the comparison of mixed pairs (RT), the IRC decreases to around 0.3 in both Experiments 2A and 2B. In this last subset, the IRC in Experiment 2A is significantly smaller than for any other (sub)set in Experiment 2A. In Experiment 2B, there is a significant difference between the dissimilarity ratings for RT and RR pairs.

### 3.3. Discussion

The clustering solution for Set 1 could be interpreted as featuring two distinct facets of timbre, namely instrument categories (or families) and continuous acoustic aspects such as sound brightness. Two of the four clusters were constituted by instruments with impulsive excitation, the other two subsumed continuously excited instruments. The two impulsive clusters differentiated themselves by spectral qualities rather than instrumental families, because the two very bright timbres, vibraphone and harpsichord, were part of one cluster. The two clusters of continuously excited instruments split into woodwinds and string instruments. This interpretation of the clustering solution suggests that multiple acoustic and categorical factors may affect musicians' dissimilarity ratings of western orchestral instruments. The last section of this manuscript attempts to model these intertwining and correlated aspects in more quantitative detail. The clustering solutions of the mixed Set 3 (Experiments 2A and 2B) exhibited two clusters of recorded tones, one cluster for transformed tones, and two mixed clusters. This means that both category membership (recordings, transformations) and acoustic similarity (e.g., brightness) appeared to act as differentiating features.

Ratings in Experiment 2A were symmetric for pairs within each set of recordings or transformations. As expected, asymmetries occurred for cross-category comparisons involving recorded and transformed tones. Pairs in which the acoustic recording was followed by the synthetic transformation generally exhibited lower dissimilarity ratings than the reverse order. This effect was replicated in a within-subjects design with a different group of musicians in Experiment 2B, and no such effect occurred for any other (sub)set. This finding suggests that sound category membership may exert an effect on dissimilarity ratings, as no simple acoustic factor can plausibly account for this effect of directionality.

To our knowledge, this is the first systematic report of asymmetries in timbre dissimilarity ratings. This effect occurred although subjects were not instructed to treat one sound as a referent and one as a subject of the comparison. Neither did we implement a directed dissimilarity rating (“How different is A to B?”), but an undirected one (“How different are A and B?”). If one assumes that auditory presentation is analogous to language, i.e., places the comparison's subject before the referent, then the direction of observed asymmetries would be opposed to what was observed for the similarities of stimuli such as countries, figures, letters, morse-code signals and integers by Tversky (1977) and Rosch (1975)—we saw that the transformation-recording pairs were generally rated less similar compared to the reverse order. The only auditory stimuli discussed by Tversky (1977) were morse-code signals, where it was assumed that longer signals act as referents and where the reported asymmetries yielded higher similarity for short-long pairs than the reverse. On that basis, it was concluded that the directionality of comparisons must be identical in the auditory domain, such that the referent follows the subject. For spectrally rich timbral stimuli, the opposite could be true, however, as the presentation of a stimulus affects processing of any stimulus presented shortly after, due to automatic stimulus-specific neural adaptation as part of sensory memory (Demany and Semal, 2007; McKeown and Wellsted, 2009). From that perspective, the second timbre is interpreted “in light of” the first, meaning the first would act as a referent. What further complicates the issue is that asymmetries only occurred systematically for cross-category comparisons, which may suggest that categorical representations independent of sensory memory are driving the effect. Note that this also leaves open the question of whether the current effect is of a perceptual nature or due to a shift in judgment strategies, commonly found in “top-down effects” (Firestone and Scholl, 2015; Storrs, 2015).

It was finally shown that inter-rater correlations (IRC) in Experiments 2A and 2B are relatively high for all pairs of timbres, except the cross-category comparisons of Set 3. This indicates that in this type of comparison, raters lost a common frame of reference. We interpret this as an index of optional processes in dissimilarity ratings (Melara et al., 1992). In the within-set comparisons of Set 1 and Set 2, comparisons may have been driven to a larger extent by sets of acoustic or categorical features similarly weighted across subjects.

Because the reduced IRCs and the rating asymmetries occurred conjointly, one may argue that one effect drove the other. It seems unlikely, though, that asymmetries were simply a coincidental artifact of a reduced IRC, given that they were reproduced in Experiment 2B in an altered design. Further research is required to better understand subjective rating behavior for timbres that have very different source origins and categorical affordances.

## 4. Dissimilarity models and analyses

The above findings on cross-category comparisons provide evidence for that categorical information may play a role in timbre dissimilarity ratings as these results seem unlikely to be explained on acoustic grounds alone. At the same time, they are based on a rather pathological comparison, namely that of familiar instrumental recordings and unfamiliar digital transformations. The question therefore becomes whether similar processes take place in the perhaps more “standard” scenario of comparing sounds from acoustic instruments. For the latter, instrument category and acoustic qualities of course coincide to a large extent (Giordano and McAdams, 2010), although not completely. Take the difference between the piano and the harpsichord or the vibraphone and marimba; the members of both pairs may feature quite different acoustic qualities although they belong to the same instrument family: keyboard and mallet instruments, respectively. Using an exploratory regression analysis, we thus set out to quantify which types of stimulus representation, acoustic or categorical, musicians took into account in their timbre dissimilarity ratings.

In the following, we first present a latent-variable-based model of acoustic timbre dissimilarity (partial least-squares regression, PLSR), well-suited to deal with collinear predictors. We then add categorical predictors to the model, which solely take into account instrument families, excitation mechanisms and types of acoustic resonators. We finally demonstrate that the highest correlations are obtained by taking into account both classes of predictors, acoustic and categorical. Note that the acoustic model will be treated in a “black-box” approach—the aim of this section is not to pin down the most parsimonious acoustic description of timbre, but for the sake of argument it must suffice to provide a robust, although potentially over-complete, acoustic model and to show that the model fit still improves with the inclusion of categorical variables.

### 4.1. Approach

We used the TimbreToolbox (Peeters et al., 2011), a large set of audio descriptors that describes the acoustic structure of audio signals with a focus on timbral qualities. We selected 34 out of its 164 descriptors, derived from measures of the temporal and spectral envelopes of the signal. The temporal envelope is computed by the Hilbert transform. Temporal descriptors focus on attack (McAdams et al., 1995) and decay properties of tones and measures of energy modulation (Elliott et al., 2013). Spectral descriptors are computed from an ERB-spaced Gammatone filterbank decomposition of the signal. They are measured for each 25-ms time frame and are summarized via the median and interquartile range as measures of central tendency and variability, respectively. Spectral descriptors include the first four moments of the spectral distribution, such as the spectral centroid that has been shown to correlate with perceived brightness (McAdams et al., 1995). Additional descriptors of the spectral distribution such as spectral slope or rolloff are included, but also measures of spectrotemporal variation, relevant to capture the perceptual dimension of *spectral flux* (McAdams et al., 1995). A full list of the descriptors is given in Table 2.

**Table 2:** **List of acoustic descriptors from the TimbreToolbox (Peeters et al., 2011)**.

**Temporal**	**Spectral**
(1) Attack duration [s]	(13) Centroid (med) [F]
(2) Decay duration [s]	(14) Centroid (IQR) [F]
(3) Release [s]	(15) Spread (med) [F]
(4) Log-attack time [log(s)]	(16) Spread (IQR) [F]
(5) Attack slope [a/s]	(17) Skew (med) [–]
(6) Decrease slope [log(a)/s]	(18) Skew (IQR) [–]
(7) Temporal centroid [s]	(19) Kurtosis (med) –
(8) Effective duration [s]	(20) Kurtosis (IQR) [–]
(9) Frequency of energy modulation [Hz]	(21) Slope (med) [F^−1^]
(10) Amplitude of energy modulation [a]	(22) Slope (IQR) [F^−1^]
(11) RMS envelope (med) [a]	(23) Decrease (med) [–]
(12) RMS envelope (IQR) [a]	(24) Decrease (IQR) [–]
	(25) Rolloff (med) [F]
	(26) Rolloff (IQR) [F]
	(27) Spectro-temporal variation (med) [–]
	(28) Spectro-temporal variation (IQR) [–]
	(29) Frame energy (med) [a^2^]
	(30) Frame energy (IQR) [a^2^]
	(31) Flatness (med) [–]
	(32) Flatness (IQR) [–]
	(33) Crest (med) [–]
	(34) Crest (IQR) [–]

*For spectral descriptors and the RMS envelope, medians (med) and interquartile range (IQR) summarize the time-varying descriptors computed over time frames of 25 ms. Square brackets provide descriptor units (a, audio signal amplitude; F, ERB-rate units). Temporal descriptors are computed from the signal energy (temporal) envelope, spectral (and spectro-temporal) descriptors from the ERB gammatone filterbank representation*.

The TimbreToolbox provided the *n* = 34 scalar descriptors for all 14 sounds. In order to obtain a predictor of acoustic dissimilarity, we computed the absolute difference of descriptor values (deltas) for each pair of sounds, yielding *m* = 105 comparisons. The final design matrix *X* (*m* × *n*) thus concatenated descriptor deltas as column vectors. The dependent variable *y* (*m* × 1) contained the 105 mean dissimilarity ratings for the respective set (averaged over the order of presentation).

In order to handle collinearity of predictors (Peeters et al., 2011), we used partial least-squares regression (PLSR; Geladi and Kowalski, 1986; Wold et al., 2001). PLSR is a regression technique that projects the predicted and observed variables onto respective sets of latent variables, such that the sets' mutual covariance is maximized. More precisely, given a dependent variable *y* and an design matrix *X*, PLSR generates a latent decomposition such that *X* = *TP*′ + *E* and *y* = *Wq*′ + *F* with loadings matrices *P* (*n* × *k*) and *q* (1 × *k*), and components (“scores”) *T* (*m* × *k*) and W (*m* × *k*) plus error terms *E* and *F*. The decomposition maximizes the covariance of *T* and *W*, which yields latent variables that are optimized to capture the linear relation between observations and predictions. For that reason, PLSR also differs from principal component analysis (PCA) followed by multivariate linear regression (MLR), which does not specifically adapt the latent decomposition to the dependent variable of interest. The regression coefficients for the original design matrix can be obtained by β = *W*(*P*′*W*)^−1^*q* (cf. Mehmood et al., 2012), which yields a link to the original MLR design via *y* = *Xβ* + *F*. In order to prevent overfitting of the response variable, the model complexity *k* is usually selected via cross-validation (Wold et al., 2001). Here we use PLSR as implemented in the plsregress.m function provided by MATLAB version R2013a (The MathWorks, Inc., Natick, MA), which applies the SIMPLS algorithm (De Jong, 1993). The significance of the individual coefficients β_*i*_ (*i* = 1, …, *n*) was estimated by bootstrapping 95% confidence intervals (percentile method) for the set of β = (_β_*i*_)*i*_ coefficients (Mehmood et al., 2012); if intervals overlapped with zero, a variable's contribution was considered to be not significant. All variables were z-normalized before entering the model.

### 4.2. Acoustic model

We opted to use a model with *k* = 2 components, which exhibited minimal 6-fold cross-validation error in the response variable compared to all other choices of *k*. This solution explained 47% of variance in the design matrix *X*. In order to first validate the general approach, we fitted models to all four dissimilarity data sets from Experiments 2A and 2B. These were tested on every other set. This evaluated the model not only on one fairly homogeneous set of sounds (as would be the case for conducting regular cross-validation on Set 1), but also allowed us to observe effects of model generalization to completely novel sets of sounds (Training: Set 1, Test: Set 3), sets in which half of the sounds are new (e.g., Train: Set 2, Test: Set 3), as well as same sets of sound but with the dependent variable stemming from a different set of participants (e.g., Train: Set 3, Experiment 2A, Test: Set 3, Experiment 2B).

Table 3 provides the proportions of explained variance (*R*^2^) in *y*. Values for each model, tested on the data to which it was fitted (i.e., the table's diagonal), range between 0.79 (Set 1, Experiment 2A) and 0.84 (Set 2, Experiment 2A). Numbers in brackets correspond to the model variant in which non-significant variables were omitted. The fact that *R*^2^ values only differ marginally between the full models and those with omitted variables indicates that these variables indeed had negligible effects on explaining the response variable. Models generalized fairly well, in particular when only the participants changed (i.e., for Experiments 2A and 2B for Set 3), but also when only half of the sounds were novel to the model. The worst generalization was for the models fitted to Set 1 or 2, evaluated on Sets 2 and 1, respectively, yielding a little less than 60% of explained variance. Overall, this demonstrates that this approach is quite robust as it explains the largest proportion of variance in the rating data on acoustic properties alone, even for models whose training sets differed from the test sets.

**Table 3:** **Variance explained (***R***^**2**^) for timbre dissimilarity models and their generalization performance across sets and experiments**.

	**Data (*y, X*)**			
**Model (β)**	**Set 1 (E2A)**	**Set 2 (E2A)**	**Set 3 (E2A)**	**Set 3 (E2B)**
Set 1 (E2A)	0.79 (0.78)	0.57 (0.57)	0.72 (0.72)	0.72 (0.70)
Set 2 (E2A)	0.58 (0.60)	0.84 (0.84)	0.68 (0.69)	0.60 (0.61)
Set 3 (E2A)	0.74 (0.73)	0.67 (0.66)	0.83 (0.84)	0.81 (0.81)
Set 3 (E2B)	0.75 (0.72)	0.58 (0.59)	0.82 (0.83)	0.83 (0.83)

*Models fitted to the four data sets (rows) from Experiments 2A, 2B, cross-validated on the same four sets (columns). Numbers in parentheses indicate performance of the reduced model for which non-significant coefficients (estimated by bootstrapping) were omitted*.

### 4.3. Including categorical variables

**Figure 5:**
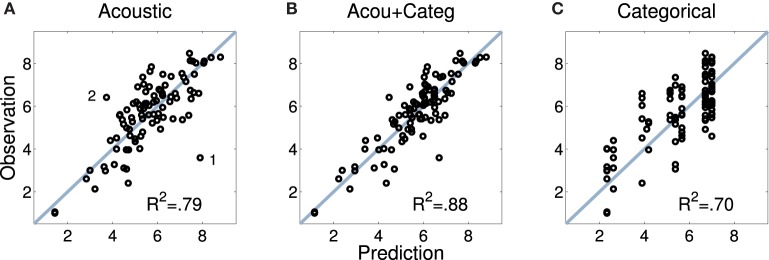
**Mean pairwise dissimilarity ratings for Set 1 (observations; y axis) and predictions based upon acoustic descriptors (A), audio and categorical predictors combined (B), and category membership of the instruments (C)**. Data points 1 and 2 in the left panel are discussed in the text.

Figure 5A displays the predicted and observed dissimilarities for the acoustic model introduced above. Although there is generally a good fit, the plot highlights two outliers (annotated as 1 and 2 in the plot). Point 1 stems from the marimba-vibraphone pair for which the acoustic model overestimated the dissimilarity rating, and point 2 from the harp-trumpet pair, for which ratings were underestimated on acoustic grounds alone. This again suggests that listeners not only based their ratings on acoustic information, but also took into account categorical information such as instrument families: Because the marimba and the vibraphone are both percussion instruments, they were rated as more similar than would be predicted given their acoustic differences. The reverse may have been at play for the harp and the trumpet, members of the string and brass families, respectively. In order to provide a quantitative footing for this intuition, we considered four additional categorical predictors of dissimilarity related to the mechanics of instruments and their families. These categories were not based on continuous acoustic descriptions of the audio signal, but may have been inferred perceptually and therefore influenced the dissimilarity ratings.

Table 4 lists all 14 instruments and their class memberships (cf. Lakatos, 2000). Here we considered categories based on two types of differences in instrument excitation (impulsive, continuous; pluck, struck, bowed, blown), resonator type (string, air column, bar), and common instrument families in the western orchestra (woodwinds, brass, strings, keyboards, percussion). For all of the four category types, dissimilarity between instruments was treated as a binary code (Giordano et al., 2012), i.e., given a 0 if members from a pair shared the same category and a 1 otherwise. The question was whether taking these variables into account would improve the model fit (given that mere overfitting was controlled for by using PLSR).

**Table 4:** **Instrumental categories based upon excitation and resonator**.

		**Resonator**		
**Excitation**		**String**	**Air column**	**Bar**
Continuous	Blown		BCL^1^, BSN^1^, FLT^1^, HRN^2^, TRP^2^	
	Bowed	VLI^4^, VCE^4^		
Impulsive	Struck	PNO^3^		VBS^5^, MBA^5^
	Pluck	VLP^4^, VCP^4^, HCD^3^, HRP^4^		

*Membership to instrument families is indicated by superscript numerals: (1) woodwinds, (2) brass, (3) keyboards, (4) string, (5) percussion*.

In order to take examples from the opposite ends of the scale, let us start with the dissimilarity of the marimba and the vibraphone. The above categorical variables would yield a zero contribution to the overall dissimilarity of this pair, because both instruments fall into the same categories for all four variables. The harp and the trumpet, on the contrary, do not share any category. By including these categorical variables in the regression model, the predicted dissimilarity of this pair would thus increase by the sum of the four variables' regression coefficients.

Categorical descriptor 1 (family) correlated significantly (*p* < 0.05) with all (!) of the other 34 acoustic descriptors with median correlations of med *r*_(103)_ = 0.28. Excitation 1 (impulsive, continuous) correlated with 18 [med *r*_(103)_ = 0.19], excitation 2 (pluck, struck, bowed, blown) with 33 [med *r*_(103)_ = 0.29], and resonator type with eight acoustic descriptors [med *r*_(103)_ = 0.11].

**Figure 6:**
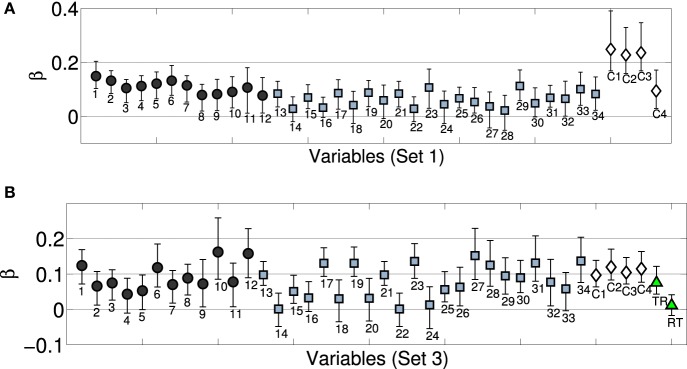
**Bootstrapped regression coefficients (standardized) for complete models (acoustic+categorical descriptors) of Set 1 (A) and Set 3 (B, depicts the model that predicts both orders of presentation)**. (Black) circles correspond to temporal envelope descriptors, (blue) squares to spectral descriptors, (white) diamonds to the four categorical descriptors (within recordings), (green) triangles (Set 3) to across sound category (rec-trans) comparisons. Enumerations of variables corresponds to Table 2. Categorical variables correspond to (C1) instrument family, (C2) excitation 1 (impulsive, continuous), (C3) excitation 2 (struck, pluck, bowed, blown), and (C4) resonator type (string, air column, bar). RT encode recording-transformation pairs, TR the reverse. Error bars correspond to bootstrapped 95% confidence intervals.

Figure 5B displays predicted and observed values for the model including the full set of acoustic and categorical variables, significantly improving the model fit by 10% as compared to the solely acoustic model (Fisher's *z* = −2.22, *p* = 0.026, two-tailed), and also visibly improving the fit for the two outliers discussed above. Notably, all categorical descriptors yield significant contributions as their (bootstrapped) confidence intervals do not overlap with zero, as highlighted in Figure 6 (white diamonds), which depicts the estimated coefficients (standardized β) for the full model. For the spectral descriptors, the majority of the inter-quartile-range descriptors appear to not provide an important contribution, whereas all but one of the median descriptors do contribute significantly. Similarly, all temporal descriptors contribute significantly. Contributions from all four categorical descriptors are significant, although differences in resonator type (encoded by the rightmost variable) are not as strongly taken into account. Moreover, the four categorical descriptors on their own (Figure 5, right), already explain 70% of the variance in the ratings (which is not significantly different from the fit of the solely acoustic model, Fisher's *z* = 1.41, *p* = 0.16, two-tailed). For this exclusively categorical model, resonator type was the only variable that failed to make a significant contribution as indicated by bootstrapped confidence intervals (not presented here).

We finally considered whether these findings would generalize to Set 3. Dissimilarity ratings for Set 3 were averaged over the order of presentation, as well as across Experiments 2A and 2B. We included the same four categorical predictors as above (although they only applied to the subset of 21 pairs among the seven acoustic recordings part of Set 3) and further added a binary variable that encoded across-category comparisons (indexing rec-trans or trans-rec pairs as 1, and all other pairs as 0). Because categorical descriptors were here construed to encode the dissimilarity based on shared features, instrument categories could not be taken into account for mixed pairs (because they are undefined for transformations). This means that for the subset of 21 pairs from the recordings, the same predictors were considered as in Set 1, but among the 21 pairs of transformations or the 49 mixed pairs, there weren't any categorical dissimilarities contributing to the regression model. In comparison to Set 1, categorical dissimilarity was therefore encoded quite coarsely. Nonetheless, all five categorical variables contributed significantly as indicated by bootstrapped confidence intervals that did not overlap with zero. The model fit increased from *R*^2^ = 0.83 for the solely acoustic model to *R*^2^ = 0.86 for the complete model, although that increase was not significant (Fisher's *z* = 0.66, *p* = 0.51, two-tailed).

In a last step, we considered the same model without averaging rating data over the order of presentation (i.e., yielding a model with 182 data points instead of 91 as above). In order to control for asymmetries in ratings of mixed pairs (see Section 3.2.2), we used two independent binary variables, one indexing the order recording-transformation (i.e., yielding 1 for rec-trans pairs, and 0 otherwise), the other encoding the reverse order (i.e., yielding 1 for trans-rec pairs, 0: otherwise), in addition to the other four categorical variables that only applied to pairs among recordings. Again, the inclusion of the categorical variables improved from *R*^2^ = 0.73 to *R*^2^ = 0.77 (although insignificantly, Fisher's *z* = 0.78, *p* = 0.42, two-tailed), and regression coefficients of all four categorical variables specific to the recordings were significantly different from zero, as indicated by bootstrapping. However, only the variable encoding the mixed pair with the order trans-rec had significant positive weight; the variable encoding the reversed order was deemed insignificant by bootstrapping. Figure 6 (bottom) displays the corresponding model coefficients.

Note that in contrast to Set 1, where categorical variables alone already explained 77% of variance in the ratings, the solely categorical model achieved a fit of *R*^2^ = 0.41 and *R*^2^ = 0.31 for Set 3 (averaged and not averaged across orders of presentation, respectively). This reflects the above mentioned coarseness of the encoding of the categorical dissimilarity for Set 3.

### 4.4. Discussion

This section described a novel model of timbre dissimilarity using partial least-squares regression. Scalar descriptors of the acoustic signal provided good predictions of timbre dissimilarity ratings, which generalized to other sets of sounds. By a *post-hoc* inclusion of a set of categorical predictors that described an instrument's family membership and facts about source and excitation mechanisms in Set 1, correlations with the observed timbre dissimilarities could be improved by around ten percentage points of the explained variance with significantly better fit compared to the solely acoustic or categorical model. Notably, these categories alone predicted around 70% of the rating variance in Set 1.

The model for Set 3 improved by 3–4 percentage points when categorical variables were added. Importantly, the model qualified the asymmetries discussed above by suggesting that only when the transformation precedes the recording does categorical information seem to strongly affect ratings, but this does not hold for the reversed order. The smaller increase in fit achieved by categorical variables for Set 3 compared to Set 1 may be attributed to the circumstance that the fine grained categorization by the four within-recordings variables only encompassed a quarter of all comparisons in Set 3, thus effectively reducing their predictive power when quantified on the basis of the full set.

Overall, we interpret these results as evidence that timbre dissimilarity ratings are informed by both continuously varying “low-level” acoustic properties, transformed into an auditory sensory representation available to the listener, as well as more “cognitive” categorical and semantic information from long-term memory inferred from the sensory representation. The regression analysis thus plausibly extends the above hypothesis on category effects in timbre dissimilarity ratings to within-set comparisons for well-known acoustic timbres from the western orchestra that can be easily associated with instrument categories. In effect, optional processes in dissimilarity ratings (Melara et al., 1992) may not only be at play in the “pathological” situation of comparing sounds with very different origins (instrumental vs. synthetic), but in any dissimilarity rating of stimuli that evokes source categories. More generally, this interpretation resonates with Tversky's comments on similarity as a complex concept. “Similarity has two faces: causal and derivative. It serves as a basis for the classification of objects, but it is also influenced by the adopted classification” (Tversky, 1977, p. 344).

It could be argued that the categorical descriptors only described acoustic and sensory aspects in a more precise way than the acoustic descriptors. However, their rough binary nature (e.g., describing attack quality by simply two categories) together with the comparatively good fit that the exclusively acoustic model achieved renders that hypothesis unlikely. This relates to the discussed experimental obstacle in this domain, namely the inherent coupling of acoustics and categories, that allows listeners to infer categories in the first place: a majority of acoustic variables correlated significantly with any of the categorical ones, making it impossible to fully disentangle sensory and cognitive aspects for natural acoustic stimuli that listeners are familiar with, i.e., for which they possess categories. However, there are exceptions to this coupling, as illustrated by the example of the marimba-vibraphone pair (within instrumental family), whose dissimilarity was overestimated on acoustic grounds, or the trumpet-harp comparison (across family) whose dissimilarity was underestimated in the solely acoustic model. A natural follow-up question then would be whether the suggested effects are under intentional subjective control, that is, whether instructing and training participants to base their ratings solely upon acoustic properties would diminish the observed effects.

Broadening the view, the current findings feature certain parallels with aspects of the literature on speech perception. For example, Zarate et al. (2015) suggested that acoustical, as well as pre-lexical phonological information, contribute to speaker identification (also see Remez et al., 2007; Obleser and Eisner, 2009). Identification performance was above chance for non-speech vocalizations, demonstrating the importance of solely acoustic information, but native English speakers' accuracy improved with increasing phonological familiarity of speech tokens (Mandarin, German, Pseudo-English, English). Again, there seems to be an interplay of basic acoustic factors and higher-level properties of speech signals that listeners need to be familiar with in order for it to become useful to them. From an even broader perspective, related observations have been made in computational music classification. For example, McKay and Fujinaga (2008) showed that combining variables extracted from the audio signal with non-acoustic types of information (e.g., symbolic MIDI data) markedly increased genre classification accuracy, again underlining the value of combining acoustic and categorical types of information representations.

## 5. Conclusion

This paper explored the role of acoustic and categorical information in timbre dissimilarity ratings. Experiment 1 provided data on the identifiability and familiarity of sounds. By means of filterbank-based sound analysis-synthesis, we created transformed tones that were generally rated as less familiar than recorded acoustic tones. We selected a subset of stimuli from the least familiar transformations that were subsequently rated on pairwise dissimilarity in Experiments 2A and 2B., along with a set of recorded acoustic tones and a mixed set. We observed that the dissimilarity data of the recorded instrument timbres clustered into subsets that distinguished timbres according to acoustic and categorical properties, such as brightness and instrument family, respectively. For the subset of cross-category comparisons in Set 3 that involved both recordings and transformations, we observed asymmetries in the distribution of ratings, as well as a stark decay of inter-rater agreement. Subsequently, these effects were replicated in a more robust within-subjects design in Experiment 2B and cannot be explained by merely acoustic factors. Note that within-set dissimilarities did not show asymmetric tendencies. In a last section we explored a novel model of timbre dissimilarity that compared the contributions of both acoustic and categorical features. The strongest correlation with the observed dissimilarities was achieved when both kinds of timbre descriptors were taken into account.

In the introduction, musical timbre was defined as a seemingly hybrid concept that encompasses both sensory and categorical components. Subsuming both facets under one term does not, consequently, constitute a lack of definitional precision, but acknowledges the multifaceted nature of information representation in the human mind. To borrow from Fuster (2003),

“Every percept has two components intertwined, the sensory-induced *re-cognition* of a category of cognitive information in memory and the categorization of new sensory impressions in the light of that retrieved memory. Perception can thus be viewed as the interpretation of new experiences based on assumptions from prior experience” (p. 84).

Our data on the interaction of acoustic and categorical facets in timbre dissimilarity suggest that the percept of timbre is a superb example of this duality. Timbre perception naturally associates a sensory representation of an acoustic waveform to hierarchically ordered categories of sound production stored in long-term memory. The listening brain represents, simultaneously, “the sound” and “the idea” of a musical instrument. Future research on timbre perception should attempt to distinguish and further disentangle these levels of representation.

## Funding

This work was supported by a grant from the Canadian Natural Sciences and Engineering Research Council (RGPIN 312774) and a Canada Research Chair (950-223484) awarded to Stephen McAdams; a Harman Scholarship from the Audio Engineering Society's Educational Foundation and an International Québec Merit Scholarship to Kai Siedenburg.

### Conflict of interest statement

The authors declare that the research was conducted in the absence of any commercial or financial relationships that could be construed as a potential conflict of interest.
